# Supplementary material for: MetaRibo-Seq measures translation in microbiomes
Source: Nat Commun. 2020 Jun 29;11:3268. doi: 10.1038/s41467-020-17081-z (PMC7324362; doi:10.1038/s41467-020-17081-z)
Supplement: Supplementary file 10 — Supplementary Data 7 [file 41467_2020_17081_MOESM10_ESM.zip › File2/Confidence_High_Taxonomy/18266_out.krona.html]

Javascript must be enabled to view this page.

members
magnitude
magnitudeUnassigned
count
unassigned
taxon
rank

18266\_out

4

superkingdom
4
2

4
1239
phylum

186801
4
class

order
4
186802

SRS043841\_contig\_number\_contig-100\_26849.26850
1

family
1
186803

1
39491

SRS078419\_contig\_number\_contig-100\_33710.68208
species

541000
1
family

genus
1263
1

species

SRS893327\_contig\_number\_contig-100\_17259.51146
1
165186

1
186806
family

genus
1
1730

species
142586
1

SRS024435\_contig\_number\_contig-100\_222.222
